# Intrinsic Up-Regulation of 2-AG Favors an Area Specific Neuronal Survival in Different In Vitro Models of Neuronal Damage

**DOI:** 10.1371/journal.pone.0051208

**Published:** 2012-12-20

**Authors:** Sonja Kallendrusch, Constance Hobusch, Angela Ehrlich, Marcin Nowicki, Simone Ziebell, Ingo Bechmann, Gerd Geisslinger, Marco Koch, Faramarz Dehghani

**Affiliations:** 1 Institut für Anatomie, Universität Leipzig, Leipzig, Germany; 2 Lipid Signaling Forschungszentrum Frankfurt, Frankfurt, Germany; 3 Institut für Pharmakologie, Goethe Universität Frankfurt,Frankfurt, Germany; 4 Institut für Anatomie und Zellbiologie, Martin Luther Universität, Halle-Wittenberg, Halle, Germany; University of Medicine and Dentistry of New Jersey, United States of America

## Abstract

**Background:**

The endocannabinoid 2-arachidonoyl glycerol (2-AG) acts as a retrograde messenger and modulates synaptic signaling e. g. in the hippocampus. 2-AG also exerts neuroprotective effects under pathological situations. To better understand the mechanism beyond physiological signaling we used Organotypic Entorhino-Hippocampal Slice Cultures (OHSC) and investigated the temporal regulation of 2-AG in different cell subsets during excitotoxic lesion and dendritic lesion of long range projections in the enthorhinal cortex (EC), dentate gyrus (DG) and the cornu ammonis region 1 (CA1).

**Results:**

2-AG levels were elevated 24 h after excitotoxic lesion in CA1 and DG (but not EC) and 24 h after perforant pathway transection (PPT) in the DG only. After PPT diacylglycerol lipase alpha (DAGL) protein, the synthesizing enzyme of 2-AG was decreased when *Dagl* mRNA expression and 2-AG levels were enhanced. In contrast to DAGL, the 2-AG hydrolyzing enzyme monoacylglycerol lipase (MAGL) showed no alterations in total protein and mRNA expression after PPT in OHSC. MAGL immunoreaction underwent a redistribution after PPT and excitotoxic lesion since MAGL IR disappeared in astrocytes of lesioned OHSC. DAGL and MAGL immunoreactions were not detectable in microglia at all investigated time points. Thus, induction of the neuroprotective endocannabinoid 2-AG might be generally accomplished by down-regulation of MAGL in astrocytes after neuronal lesions.

**Conclusion:**

Increase in 2-AG levels during secondary neuronal damage reflects a general neuroprotective mechanism since it occurred independently in both different lesion models. This intrinsic up-regulation of 2-AG is synergistically controlled by DAGL and MAGL in neurons and astrocytes and thus represents a protective system for neurons that is involved in dendritic reorganisation.

## Introduction

The endocannabinoid (eCB) system was shown to be involved in processes of neurodegeneration. Increased concentrations of eCBs such as 2-arachidonoylglycerol (2-AG) were found after different brain injuries [1] and various cannabinoids protected against ischemia, excitotoxicity or traumatic brain injury (TBI) [2], [3], [4], [5]. Furthermore, the involvement of the eCB system in neurodegenerative diseases, like MS, Parkinson's, Huntington's and Alzheimer's disease is more and more unraveled [6], [7], [8], [9].

Excitotoxic neuronal damage is reduced by 2-AG acting as a retrograde neurotransmitter decreasing neuronal firing at glutamatergic synapses [10]. Affecting different cannabinoid receptors and down-regulating the NFkB pathway 2-AG is involved in analgesia and anti-inflammatory processes [4], [5], [11], [12], [13]. The cannabinoid receptors type 1 (CB_1_) and type 2 (CB_2_) are the main molecular targets of 2-AG [14], [15]. CB_1_ is the most abundant G protein-coupled receptor in the brain and resides at the pre-synaptic side of neuronal synapses [16], whereas CB_2_ is mainly situated on immunoregulatory cells, like microglial cells [17], [18]. There are several reports in the literature indicating further not yet cloned cannabinoid receptors that might be activated by 2-AG as well [5], [19]. 2-AG is synthesized on demand from membrane glycerophospholipids by specific enzymes. Sn-1-diacylglycerol lipase alpha (DAGL) is the primary biosynthetic enzyme of 2-AG, found at the post-synapse of neurons [20]. Endocannabinoid signaling is further controlled by enzymatic hydrolysis and the principal 2-AG hydrolyzing enzyme is monoacylglycerol lipase (MAGL) being ascribed for 85% of total 2-AG break down [21], [22].

However, so far little is known about the contribution of different glial cell populations to 2-AG regulation and the time course of its induction. Moreover, the precise regional control of 2-AG in different brain areas still remains unknown. Therefore, we here compared the induction of the 2-AG regulatory machinery in an *in vitro* model of Organotypic Hippocampal Slice Cultures (OHSC) and investigated the temporal changes in 2-AG levels and its main enzymes DAGL and MAGL after 1) long-range projection damage as it occurs in stroke by transection of the perforant pathway and 2) excitotoxic lesion as a central mechanism of neuronal damage by application of NMDA. The excitotoxic lesions were performed with low (10 µM) and high (50 µM) NMDA doses to verify how sensitive the 2-AG machinery responds to an excitotoxic insult.

The perforant pathway connects two evolutionary distinct brain regions, namely the entothinal cortex (EC) and the outer molecular layer of the dentate gyrus (DG) and its transection allows the study of cellular and inflammatory responses not only at the lesion site but also of anterograde projection areas [23], [24], [25], [26], [27]. In the model of excitotoxicity the vulnerability of different areas within the OHSC can be analyzed. The regulation of 2-AG levels and the main synthesizing and hydrolyzing enzymes were assessed up to 72 hours after perforant pathway transection (PPT) and excitotoxicity in OHSC. Moreover, the respective cell types being responsible for 2-AG metabolism, namely neurons, astrocytes or microglia were studied.

## Materials and Methods

### Ethics Statement

All animal experiments have been approved by the ethics committees of the German federal state of Saxonia and were performed in accordance with the Policy on Ethics and the Policy on the Use of Animals in Neuroscience Research as approved by the directive 2010/63/EU of the Council of the European Union on the protection of animals used for scientific purposes. Animals were maintained under a standard and a 12∶12 hr light/dark schedule and constant temperature with food and water available *ad libitum* for the female rat. All efforts were made to minimize suffering.

### Organotypic Hippocampal Slice Cultures (OHSC)

8-day old Wistar rats were used to prepare OHSC. After decapitation the brains were dissected under aseptic conditions and transferred to 4°C minimal essential medium (MEM; Gibco BRL Life Technologies, Eggenstein, Germany) containing 1% (v/v) glutamine (Gibco) (Stoppini et al. 1991). With a sliding vibratome (Leica VT 1200 S, Leica Microsystems AG, Wetzlar, Germany) brains were horizontally cut in 350 µm-thick slices. After dissection of the hippocampus the slices were immediately placed on cell culture inserts (pore size 0.4 µm; Millipore, Schwalbach/Ts., Germany) in 6-well culture dishes (Falcon, BD Biosciences Discovery Labware, Bedford, MA) containing 1 ml slice culture medium (50% (v/v) MEM, 25% (v/v) Hanks' balanced salt solution (HBSS, Gibco), 25% (v/v) normal horse serum (NHS, Gibco), 2% (v/v) glutamine, 1.2 mg/ml glucose (Braun, Melsungen, Germany), 0.1 mg/ml streptomycin (Sigma-Aldrich, Deisenhofen, Germany), 100 µg/ml penicillin (Sigma-Aldrich), 0.8 µg/ml ascorbic acid (Sigma-Aldrich) and 1 µg/ml insulin (Boehringer, Mannheim, Germany), pH 7.4) per well. The slice cultures were kept at 35°C in a fully-humidified atmosphere with 5% (v/v) CO_2_ for 6 days until lesion was set. The culture medium was changed every other day.

### Primary cell culture

Primary cell cultures of microglial cells and astrocytes were isolated from cerebral cortices of neonatal Wistar rats (P1). The meninges were removed and the cerebral cortices were dissociated in Ca^2+^/Mg^2+^-free HBSS, containing trypsin (4 mg/ml, Boehringer) and DNAse (0.5 mg/ml, Worthington, Bedford, MA, USA). Tissue culture flasks (75 cm^2^, Falcon) were beforehand coated with poly-L-lysine (Sigma-Aldrich) and contained cell culture media (DMEM (Gibco), 4.5 g/L glucose (Gibco), 10% (v/v) fetal bovine serum (FBS, Gibco), 1% (v/v) glutamine, 100 µg/ml penicillin and 100 µg/ml streptomycin) when cells were added and cultured until further use. Microglial cells were separated from the astrocytic monolayer and were subsequently plated into 24-well dishes coated with poly-L-lysine with cell culture medium containing 2% (v/v) FBS. Astrocyte cultures were also transferred one day prior to experiments into 24-well dishes coated with poly-L-lysine. Microglia and astrocytes were treated with 10 ng/ml lipopolysaccharide (LPS) for 4 hours (h).

For primary cultures of hippocampal neurons a modified method described by Brewer and colleagues was used [28]. Briefly, Wistar rats P0 were used and brains were dissected quickly. The meninges were removed and the brains were placed into an ice-cold HBSS solution. Subsequently the hippocampi were dissected and incubated in Neurobasal Medium (Gibco) containing BSA (Sigma-Aldrich) and papain (1 mg/ml, Sigma-Aldrich) for 20 min at 37°C. Neurons were isolated by dissociation and cultured onto poly-L-lysine-coated coverslips in 24-well dishes in Neurobasal medium supplemented with B-27 (Gibco), GlutaMAX (Gibco) and penicillin/streptomycin at 37°C in a humidified atmosphere with 5% (v/v) CO_2_ for 2 weeks.

### Excitotoxicity lesion

The OHSC were randomly divided into different treatment groups and were treated according to the following treatment protocol:

CTL: Unlesioned OHSC (n = 5) were kept in culture medium for 9 days in vitro (div) without any treatment and served as controls.

JZL184: Unlesioned OHSC (n = 6) were kept in culture medium until 6 div. At 6 div the culture medium was supplemented with 1 µM JZL 184 until 9 div.

NMDA: OHSC were kept in culture medium for 6 days, lesioned with NMDA (50 µM; n = 8, Sigma-Aldrich) for 4 hours and maintained in culture medium until 9 div.

NMDA+JZL184: OHSC were lesioned at 6 div with NMDA (50 µM; n = 13, Sigma-Aldrich) for 4 h and kept in culture medium supplemented with JZL 184 (1 µM) until 9 div.

#### OHSC treatment and 2-AG measurement

In an additional set of experiments OHSC were treated with low (10 µM) or high (50 µM) NMDA concentrations for 4 h and were further maintained in slice culture medium for 24 h or 72 h post lesion (hpl). Controls were cultured with slice culture medium until 4 h, 24 h and 72 h, simultaneously with the NMDA treated OHSC. At 4 h, 24 h and 72 h samples were collected and processed for immunohistochemistry or LC-MS/MS as described below.

### Perforant Pathway Transection (PPT)

The PPT was mechanically set at 6 div by using a disposable ophthalmic scalpel with a stainless steel blade (Feather, Osaka, Japan). A binocular (Zeiss, Jena, Germany) was used to performed PPT within OHSC. The cut was set through the perforant pathway following the sulcus between the hippocampus and the EC [29]. Dissection of the EC, the DG and the cornu ammonis 1 (CA1) region was done 0, 1, 6, 12, 24, 48 or 72 hpl. Similar regions of three OHSC were pooled and shock frozen in liquid nitrogen. The tissues were stored at −80°C and processed not exceeding three days.

### LC-MS/MS

#### Sample Extraction

Lipid extraction has been carried out as previously described [30], [31]. Briefly, 70 µl ice cold H_2_O was added to the sample prior to homogenation by a mixer mill 400 (Retsch, Haan, Germany) for 90 sec at 25 Hz. Next, from the homogenate 20 µl was collected to determine the beta-actin content by Western Blot analysis (see below). The remaining 50 µl of the homogenized tissue samples were then further processed together with 25 µl internal standard (IS), 50 µl ethylacetate/n-hexan and 50 µl H_2_O. After 3 min at 10,000 rpm the samples were centrifuged and the organic phase was collected, the extraction procedure was repeated twice. Under nitrogen the ethylacetate phases evaporated and the eCB were assimilated in 25 µl acetonitril in glass vials. 10 µl were injected into an LC-MS/MS system (API, 5000, AB SCIEX, California, USA). Quality standards were always extracted with the samples for accuracy.

#### Determination of endocannabinoid concentrations

2-AG and 1-AG were analyzed from the reconstituted samples. Deuterated analytes 2-AG-d_5_, 1-AG-d_5_ were used as IS. Under gradient conditions using a Luna HST C18 (2) column (100 mm L×2 mm ID, 2.5 µm particle size; Phenomenex, Aschaffenburg, Germany) HPLC analysis was performed. An API 5000 triple quadrupole mass spectrometer with a Turbo V source (Applied Biosystems, Darmstadt, Germany) in the negative ion mode was used to perform MS and MS/MS. For 2-AG the precursor-to-product ion transitions m/z 377→303 was applied and m/z 382→303 for the deuterated 2-AG-d_5_ and 1-AG-d_5_ for the multiple reaction monitoring (MRM) with a dwell time of 70 msec respectively. The Analyst software (version 1.4; Applied Biosystems) was used to evaluate concentrations of calibration standards, quality controls and samples. <15% over the range of calibration were accepted as intra-day and inter-day variations in accuracy.

### Semi-quantitative real time PCR (rtPCR)

The site specific and time dependent changes in enzymes, DAGL and MAGL were analyzed by quantitative rtPCR. Tissue was harvested and stored in RTL*later* (Qiagen) at −80°C until further use. Total RNA was extracted from the cells using TRIzol reagent (Invitrogen) according to the manufacturer's instructions. RNA was measured and controlled for its purity by the A260/280 ratio. Total RNA was immediately reversely transcribed (Superscript II, Invitrogen) with oligo(dT) primer cDNA. 50 ng of total RNA was subjected to rtPCR using Platinum-SYBR Green® qPCR Supermix (Invitrogen). Oligonucleotide primers (Tab. S1) were designed with the Primer3 software to flank intron sequences. All primers were synthesized by MWG Biotech (Eversberg, Germany) and diluted to 10 pmol/µl. Quantitative rtPCR was performed by an CFX9 (Bio Rad laboratories, München, Germany) using the following protocol: 3 min at 95°C and 40 cycles of 10 s at 95°C and 30 s at 61°C. A product melting curve was recorded to confirm the presence of a single amplicon. The correct amplicon size and identity were additionally confirmed by agarose gel electrophoresis. Standard curves with serial dilutions of cDNA were generated for each primer pair to assert linear amplification. Threshold cycle (*C_t_*) values were set within the exponential phase of the PCR. After normalization to rodent glyceraldehyde 3-phosphate dehydrogenase (GAPDH) primer, Δ*C_t_* values were used to calculate the relative expression levels.

### Western Blot analysis

Tissue was collected and immediately stored in lysis buffer at −80°C until further use. Protein was extracted in lysis buffer containing 80 mM Tris, 70 mM SDS, 0,3 M Saccharose, 3 mM sodium orthovanadate and 0.5 mM phenylmethylsulfonyl floride (PMSF) at pH 7.4. Five µg protein were loaded onto a 7,5% or 12,5% (w/v) sodium dodecylsulfate–polyacrylamide gel. Proteins were electrotransferred to nitrocellulose membranes and non-specific protein-binding sites were blocked for 1 h with 5% (w/v) milk (Carl Roth, Karlsruhe, Germany) or 5% (v/v) Roti-block solution (Carl Roth, Karlsruhe, Germany) in TBST. Primary antibodies were diluted in 5% (w/v) milk or 5% (v/v) Roti block in TBST and membranes were incubated over-night. The following primary antibodies were used: rabbit polyclonal antibody against human DAGL (diluted 1∶2000, Frontier Science, cat.-no DGLa-Rb-Af380), rabbit polyclonal antibody against human MAGL (diluted 1∶1000; developed by K. Mackie) and mouse monoclonal antibody directed against human beta-actin (diluted 1∶40000; cat.-No A1978, Sigma-Aldrich). Membranes were washed and the secondary horse radish peroxidase-conjugated antibodies were applied for 1 h (anti-rabbit, 1∶1000; cat.-No PI 1000, Vektor laboratories, Burlingame, CA; anti-mouse IgG, 1∶4000; cat.-No CP01, Millipore, Billerica, USA). The signal of bound antibody was visualized by enhanced chemiluminescence (ECL detection system, Millipore) with radiographic films (Kodak, Stuttgart, Germany). ImageJ image analysis software (version 1.43, imagej.nih.gov/ij/download/) was used for semiquantification of the immunoreactive bands. Preabsorption with the corresponding blocking peptide was used to control the specificity of the MAGL antibody (Fig. S1A).

### Immunohistochemistry

For 4 h OHSC were fixed in 4% (w/v) PFA, washed and incubated in 15% (w/v) sucrose before sectioning (14 µm) on a cryostat 3050 S (Leica, Wetzlar, Germany). After blocking unspecific bindings by normal goat serum (1∶20 in PB saline (PBS)/Triton) for 30 min primary antibodies were added for 16 h in PBS/Triton containing 0.5% (w/v) bovine serum albumin: DAGL (diluted 1∶200, Frontier Science, cat.-no DGLa-Rb-Af380), MAGL (diluted 1∶200, Cayman Chemicals, cat no. 100035, Fig. S1B), Vglut1 (diluted 1∶1000, synaptic systems, cat no. 135311), NeuN (diluted 1∶200; cat.-No MAB377, Millipore), GFAP (diluted 1∶200; cat. No 556330 BD Pharmingen) and biotinylated isolectin B_4_ (IB_4_) (diluted 1∶50; cat.-No FL-1201 Vector Laboratories).

Triple-immunofluorescence staining was done using the primary antibodies against DAGL or MAGL combined with IB_4_ and either with NeuN or GFAP respectively. Sections were washed in PBS prior to application of the secondary antibodies Alexa flour dye 568 (1∶500, goat-anti rabbit IgG) and Alexa fluor dye 633 (1∶100, goat anti-mouse IgG; Invitrogen, Karlsruhe, Germany) for 1 h. The sections were coverslipped with Dako fluorescent mounting medium (Dako) and visualized by a Zeiss LSM 510 Meta confocal laser scanning system equipped with helium neon lasers (543 and 633 nm excitation lines) and an argon laser (488 nm excitation line).

### Propidium Iodide (PI) labeling and confocal laserscanning microscopy

Two hours prior to fixation 5 µg/ml PI was added to the slice culture medium. OHSC were then washed in PB and mounted with DAKO fluorescent mounting medium (DAKO Diagnostika GmbH, Hamburg, Germany) and were analyzed with a Zeiss confocal laser scanning microscope using the helium neon laser 543 (LSM 510, Zeiss, Gottingen, Germany). With 160-fold magnification images were obtained using the Z-mode of the confocal microscope, optically cutting the OHSC in 2 µm-thick sections. Images were then converted into a binary image and Image J software (version 1.43, imagej.nih.gov/ij/download/) was used for PI-positive neuronal nuclei counting throughout the granule cell layer (GCL) of the dentate gyrus. To correct for inter experiment variances the different conditions were normalized to the respective NMDA condition.

### siRNA transfection

MAGL gene silencing was carried out using siRNA (target sequence: 5′-CAGCGTGCTGTCTCGGAACAA-3′; sense strand: 5′-GCGUGCUGUCUCGGAACAATT-3′; antisense strand: 5′-UUGUUCCGAGACAGCACGCTG-3′; cat. no.: SI03067736, Qiagen, Hilden, Germany) and GeneSilencer siRNA transfection reagent (Genlantis-BioCat, Heidelberg, Germany) in a 6-well-culture plate. AllStars Negative Control siRNA (Qiagen) was used as a negative control. Transfection was performed according to manufacture's instructions at 5 div for 24 h before changing to native culture medium.

### Statistical Analysis

Data from at least three independent experiments were expressed as mean values (+standard error of the mean (SEM) and statistically analyzed using one way ANOVA followed by Bonferroni posttests. Analysis was conducted with Graph Pad Prism software 5 (GraphPad software, La Jolla, USA) and p<0.05 was considered significant. Gene regulation was statistically evaluated by subjecting the ΔΔ*C_t_* values derived from each preparation samples to one way ANOVA.

## Results

### 2-AG levels are up-regulated after excitotoxic lesion and PPT in areas of severe damage

**Figure 1:**
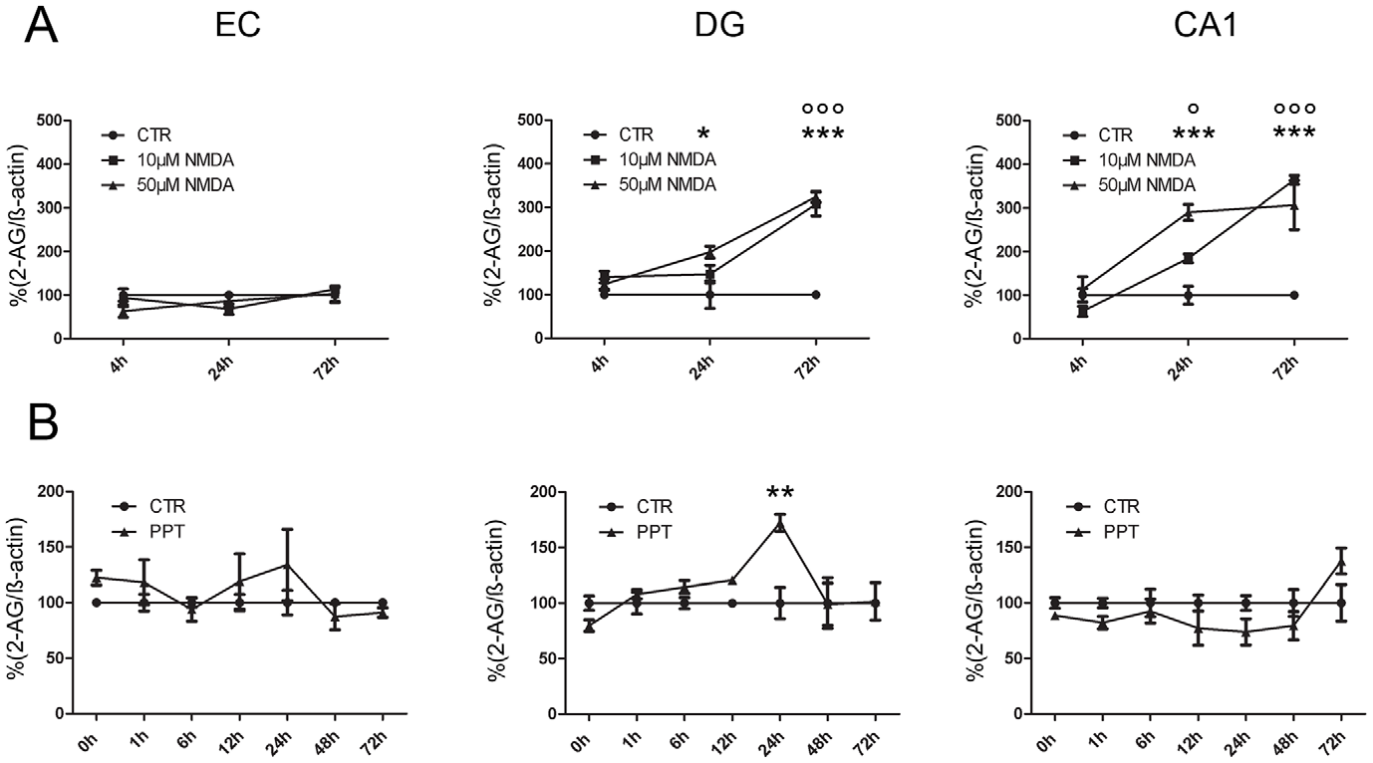
Intrinsic 2-AG regulation after neuronal damage - excitotoxic NMDA lesion and PPT. 2-AG levels in EC, DG and the CA1 region normalized against ß-actin immunosignals. Control condition (CTR) of each time-point was set as 100% (black cycles). (A) 2-AG levels after application of 10 µM and 50 µM NMDA. In the DG an increase in 2-AG was seen 24 hpl after 50 µM of NMDA. After 72 hpl 2-AG levels were elevated significantly independent of the NMDA concentration. In the CA1 region increased 2-AG levels were measured 24 and 72 hpl after 10 µM and 50 µM NMDA treatment (n = 3, °sig. for 10 µM NMDA, *sig. for 50 µM NMDA, °p<0.05, °°° or ***p<0.001). (B) 2-AG levels after PPT. In EC 2-AG levels (gray circles) did show a non significant alteration at 1 hpl in comparison to the respective CTR. In the DG, 2-AG was significantly increased 24 hpl (n = 4, **p<0.01) and in the CA1 region 2-AG levels did not differ significantly from CTR values (p>0.05).

Raw values of 3 pooled OHSC (EC, DG or CA1) contained about 3–5 ng/ml 2-AG under control conditions. The obtained 2-AG values were corrected for their ß-actin content and were then given in percent values of the respective controls. The NMDA and PPT data was expressed in relation to their time controls, respectively (Fig. 1).

#### Excitotoxic lesion

Excitotoxic NMDA lesion and dendritic denervation led to an intrinsic up-regulation of 2-AG levels in specific brain regions, namely the DG and the CA1 region after excitotoxic lesion with low and high NMDA doses (NMDA, 10 µM, 50 µM, Fig. 1A). In the EC 2-AG levels remained at control levels after excitotoxic lesion with 10 µM and 50 µM NMDA until 72 hpl (Fig. 1A). In the DG 10 µM NMDA increased 2-AG levels 72 hpl (307.50%; p<0.001) but not at 4 hpl and 24 hpl (Fig. 1A). 50 µM NMDA increased 2-AG levels at 24 hpl (197.60%) and 72 hpl (323.90%; p<0.001, Fig. 1A). 2-AG levels of the CA1 region treated with 10 µM NMDA showed a significant increase at 24 hpl (184.70%; p<0.05) and 72 hpl (364.30%; p<0.001, Fig. 1A). After treatment with 50 µM NMDA 2-AG was enhanced at 24 hpl (290.20%; p<0.001) and 72 hpl (306.70%; p<0.001, Fig. 1A). Single values of each group are presented in Table 1.

**Table 1:** Relative changes in 2-AG levels.

EC (NMDA)	2-AG (%)		DG (NMDA)	2-AG (%)		CA1 (NMDA)	2-AG (%)	
	10 µM	50 µM		10 µM	50 µM		10 µM	50 µM
4 hpl	94; p>0.05	63; p>0.05	4 hpl	140; p>0.05	124; p>0.05	4 hpl	63; p>0.05	114; p>0.05
24 hpl	68; p>0.05	87; p>0.05	24 hpl	147; p>0.05	198; p<0.01	24 hpl	185; p>0.05	290; p<0.001
72 hpl	114; p>0.05	103; p>0.05	72 hpl	308; p<0.001	324; p<0.001	72 hpl	364; p<0.001	307; p<0.001

EC (PPT)	2-AG (%)	DG (PPT)	2-AG (%)	CA1 (PPT)	2-AG (%)			
0 hpl	122; p>0.05	0 hpl	79; p>0.05	0 hpl	89; p>0.05			
1 hpl	118; p>0.05	1 hpl	107; p>0.05	1 hpl	82; p>0.05			
6 hpl	94; p>0.05	6 hpl	114; p>0.05	6 hpl	93; p>0.05			
12 hpl	119; p>0.05	12 hpl	120; p>0.05	12 hpl	77; p>0.05			
24 hpl	134; p>0.05	24 hpl	172; p<0.01	24 hpl	74; p>0.05			
48 hpl	87; p>0.05	48 hpl	98; p>0.05	48 hpl	80; p>0.05			
72 hpl	91; p>0.05	72 hpl	101; p>0.05	72 hpl	138; p>0.05			

#### Perforant Pathway Transection (PPT)

After PPT no significant changes of 2-AG levels were observed in the EC an CA1 region (Fig. 1B). In the DG 24 hpl 2-AG levels were significantly increased compared to controls (24 hpl, 172.20%; p<0.01, Fig. 1B). Thereafter 2-AG levels declined to control levels (Fig. 1B). Single values of each group are presented in Table 1.

### Transcriptional dynamics of Dagl and Magl after PPT

Semi-quantitative real time PCR was used to investigate the intrinsic regulation of (*Dagl*) and (*Magl*) enzymes of 2-AG in the different regions over time.

**Figure 2:**
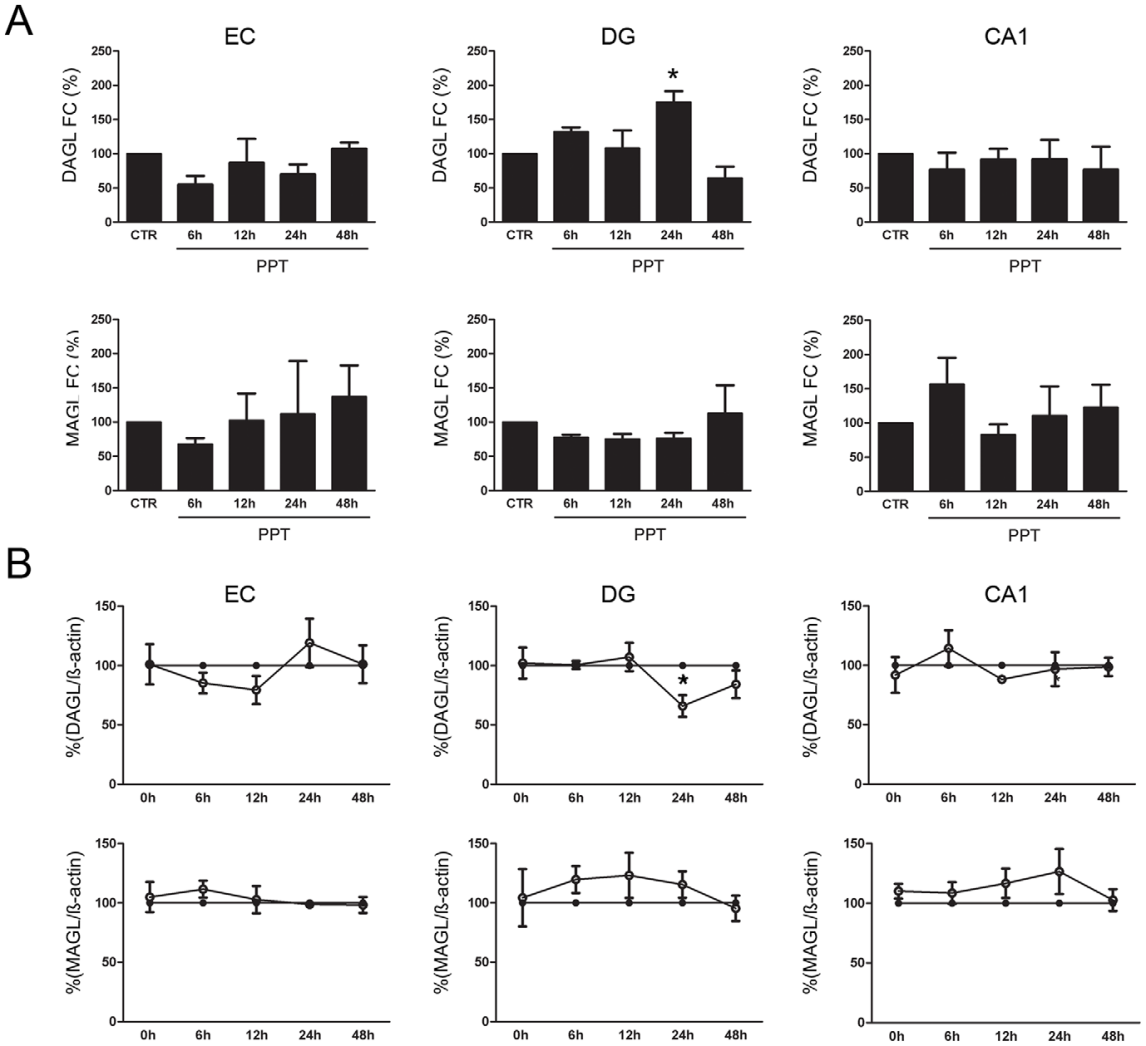
Enzymatic regulation of 2-AG after PPT. (A) *Dagl* and *Magl* mRNA regulation after PPT in the entorhinal cortex and the hippocampus after PPT. Dagl was increased after 24 hpl of PPT in the DG. (n = 3, *p <0.05) (B) DAGL (open cycles) and MAGL (open cycles) protein regulation after PPT in the entorhinal cortex and the hippocampus after PPT. DAGL was decreased after 24 hpl of PPT in the DG compared to the respective control (closed cycles). (n = 3, *p<0.05).

In the EC and CA1 regions no changes of both mRNAs were observed after PPT. *Dagl* and *Magl* expression were not significantly different compared to the control OHSC (Fig. 2A). In the DG *Dagl* expression was significantly enhanced 24 hours after PPT (24 hpl, 175.40%; p<0.05, Fig. 2A) related to the respective controls. *Magl* expression did not show significant changes (Fig. 2A

### Dynamics in DAGL and MAGL protein amount after PPT

Western Blot analysis was used to investigate the intrinsic regulation of DAGL and MAGL in different regions over time. The respective time controls were set to 100%. No changes in DAGL and MAGL were observed in the EC and CA1 regions after PPT as compared to the time control OHSC (Fig. 2B). After 24 hpl DAGL protein was significantly decreased In the DG after PPT (Fig. 2B, 24 hpl, 65.90%; p<0.05) related to the respective controls. MAGL expression in the DG did not show significant changes (Fig. 2B). Single values of each group are presented in Table 2.

**Table 2:** Relative changes in protein and mRNA levels after PPT.

EC					
Protein (%)			mRNA (%)		
Group	DAGL	MAGL	Group	DAGL	MAGL
0 h	101, p>0.05	105, p>0.05	CTR	100, p>0.05	100, p>0.05
6 h	85, p>0.05	112, p>0.05	6 h	55, p>0.05	68, p>0.05
12 h	79, p>0.05	103, p>0.05	12 h	87, p>0.05	103, p>0.05
24 h	119, p>0.05	99, p>0.05	24 h	70, p>0.05	112, p>0.05
48 h	101, p>0.05	98, p>0.05	48 h	107, p>0.05	137, p>0.05

DG					
Protein (%)			mRNA (%)		
Group	DAGL	MAGL	Group	DAGL	MAGL
0 h	102, p>0.05	104, p>0.05	CTR	100, p>0.05	100, p>0.05
6 h	101, p>0.05	120, p>0.05	6 h	132, p>0.05	78, p>0.05
12 h	107, p>0.05	123, p>0.05	12 h	108, p>0.05	76, p>0.05
24 h	66, p<0.05	115, p>0.05	24 h	175, p<0.05	76, p>0.05
48 h	84, p>0.05	95, p>0.05	48 h	65, p>0.05	113, p>0.05

CA1					
Protein (%)			mRNA (%)		
Group	DAGL	MAGL	Group	DAGL	MAGL
0 h	92, p>0.05	110, p>0.05	CTR	100, p>0.05	100, p>0.05
6 h	114, p>0.05	109, p>0.05	6 h	77, p>0.05	157, p>0.05
12 h	88, p>0.05	117, p>0.05	12 h	92, p>0.05	83, p>0.05
24 h	97, p>0.05	130, p>0.05	24 h	92, p>0.05	111, p>0.05
48 h	99, p>0.05	103, p>0.05	48 h	77, p>0.05	123, p>0.05

### Cellular distribution of DAGL and MAGL in primary neurons, microglia and astrocyte cultures

**Figure 3:**
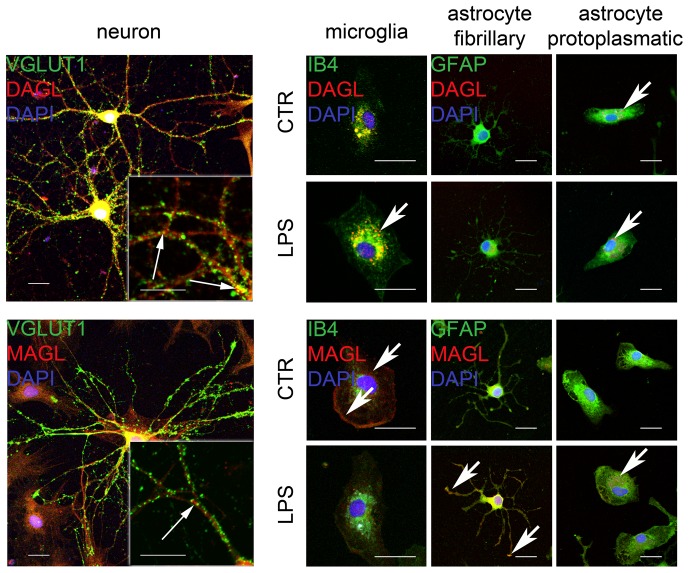
Immunocytochemical analyses of DAGL and MAGL in primary neuronal (left column), microglial (middle column) and astrocyte (left columns) cell cultures. In neurons, DAGL was distributed in the perikarya and in neuronal processes. VGLUT1 was co-localized with DAGL suggesting a close surveillance of glutamate by DAGL (arrows). MAGL was distributed in primary neuronal cultures but displayed no overlap with VGLUT1 (arrow). **Microglial cells** were labeled with DAGL in their cell bodies after LPS treatment. DAGL expression was stronger than in controls (arrow). whereas, MAGL expression in microglia was clearly visualized under control condition around the nucleus and at the outer edges (arrows) of the cells the labeling disappeared under LPS treatment (arrow). **Astrocytes** were not found immunoreactive for DAGL in fibrillary astrocytes under control condition, however DAGL was weakly induced after LPS treatment. Protoplasmic astrocytes displayed a robust DAGL immunoreaction in controls and after LPS stimulation as visualized in droplets throughout the cell body (arrows). MAGL was induced in both fibrillary and protoplasmic astrocytes after LPS treatment (arrows). Bar = 20 µm.

In isolated primary neuronal cultures DAGL immunoreaction (IR) was observed within neuronal perikarya and processes. The double staining of DAGL and VGLUT1 showed punctual colocalizations (Fig. 3). MAGL IR was not observed to be co-localized with VGLUT1 in primary neuronal culture.

In primary microglia cultures DAGL IR was located in the cell body and became more intense after LPS treatment (Fig. 3). DAGL IR was differentially distributed in astrocyte subtypes: whereas fibrillary astrocytes showed a very weak DAGL IR after LPS treatment only, protoplasmic astrocytes displayed a few punctual immunoreactions spread throughout the cell body under both, control and LPS treated conditions (Fig. 3). In IB_4_ positive microglia, MAGL IR was found in the cytoplasm and at the cell borders. Interestingly MAGL IR was very weak after LPS treatment (Fig. 3). Fibrillary astrocytes displayed MAGL IR within their cell bodies. In LPS treated cells, MAGL IR was seen in the cell bodies and to a minor extent in their processes. The IR of MAGL in protoplasmic astrocytes was very weak, no alteration was observed between the control and the LPS treated condition.

### Distribution of DAGL and MAGL in OHSC

Localizations of DAGL and MAGL IR were investigated by immunohistochemical staining with NEUN, GFAP and IB_4_ in sections obtained from OHSC at 1 hpl, 6 hpl, 12 hpl and 24 hpl.

#### Perforant pathway transaction

**Figure 4:**
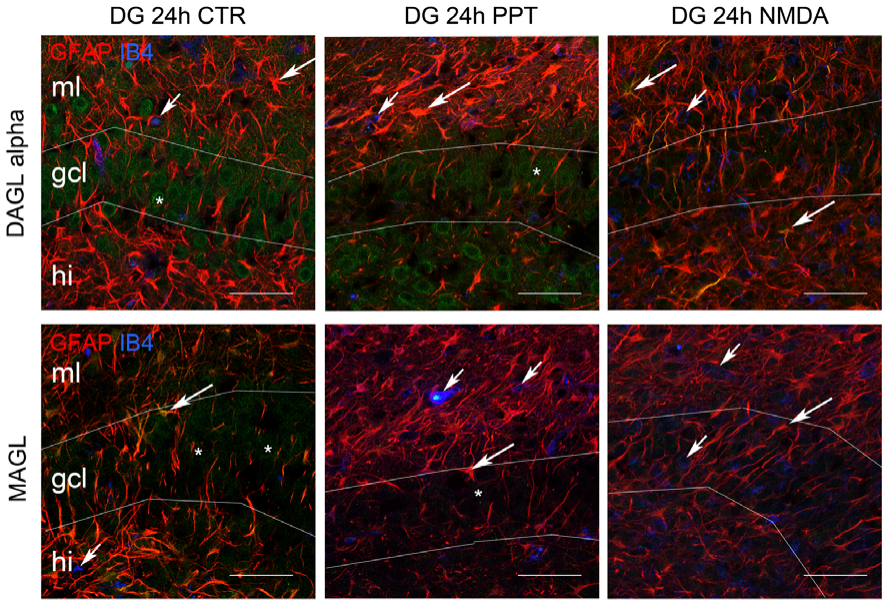
DAGLα and MAGL immunoreaction in OHSC 24 h after PPT. Images show the molecular layer (ml), the granular cell layer (gcl) and part of the hilus (hi) region. The enzymes DAGL and MAGL are given in green, GFAP in red and IB_4_ positive microglial cells are displayed in blue. The asterisks indicate NeuN co-localized DAGL and MAGL staining in the gcl (NeuN data not shown). DAGL was co-localized with GFAP positive cells only after PPT and NMDA excitotoxic lesion but not in control OHSC (big arrows). IB_4_ positive microglial cells were not DAGL immunoreactive under control conditions or after neuronal damage (small arrows). After PPT the DAGL was seen in the outer half of the gcl whereas the inner half of the granular cell layer was not labeled (24 hpl, asterisk). GFAP positive cells expressed a strong co-localization with the MAGL protein in control OHSC that disappeared 24 h after PPT and in NMDA lesioned OHSC (big arrows). A weak MAGL immunoreaction was found in vesicular structures in microglial cells presumably representing phagocytosis bodies in their cytoplasms (small arrows). (Bar = 50 µm).

DAGL IR was found in the cytoplasm of NeuN positive cells in control OHSC and in OHSC after PPT. Co-localization of DAGL IR with GFAP IR in astrocytes and IB_4_ positive microglial cells was hardly detectable in control OHSC and no change was found in OHSC after PPT until 12 hpl (not shown). After 24 hpl the DAGL IR remained in the cytoplasm of neurons and a weak DAGL IR was seen in astrocytes. Reorganization of DAGL IR was also observed 24 hpl, the tight organization of the granular cell layer dispersed and DAGL IR was found in the outer third but vanished in the inner half of the granular layer (Fig. 4). In the hilus region of some PPT lesioned OHSC enhanced DAGL IR was seen, however this finding was not consistent in all PPT lesioned OHSC.

MAGL IR showed a punctual distribution in NeuN positive cells (Fig. 4). GFAP positive astrocytes showed a strong co-localization with the MAGL IR under control conditions. Microglial cells displayed only weak or no MAGL IR in OHSC (Fig. 4). Over time, microglial cells did not show a change of MAGL IR in OHSC. At 24 hpl (PPT) MAGL IR was lower in GFAP positive cells, no other changes in cellular distribution were observed over time (Fig. 4).

#### Excitotoxic lesion

DAGL IR was found in the cytoplasm of NeuN in OHSC after excitotoxic NMDA lesion and disappeared 24 hpl. After NMDA lesion DAGL IR was mainly found in astrocytes and no longer in neuronal cytoplasm (Fig. 4).

MAGL IR was located in GFAP positive astrocytes under control conditions and could not be seen after 24 hpl (NMDA) in GFAP positive cells (Fig. 4).

### Intrinsic up-regulation of 2-AG after MAGL inhibition by JZL184 in OHSC

**Figure 5:**
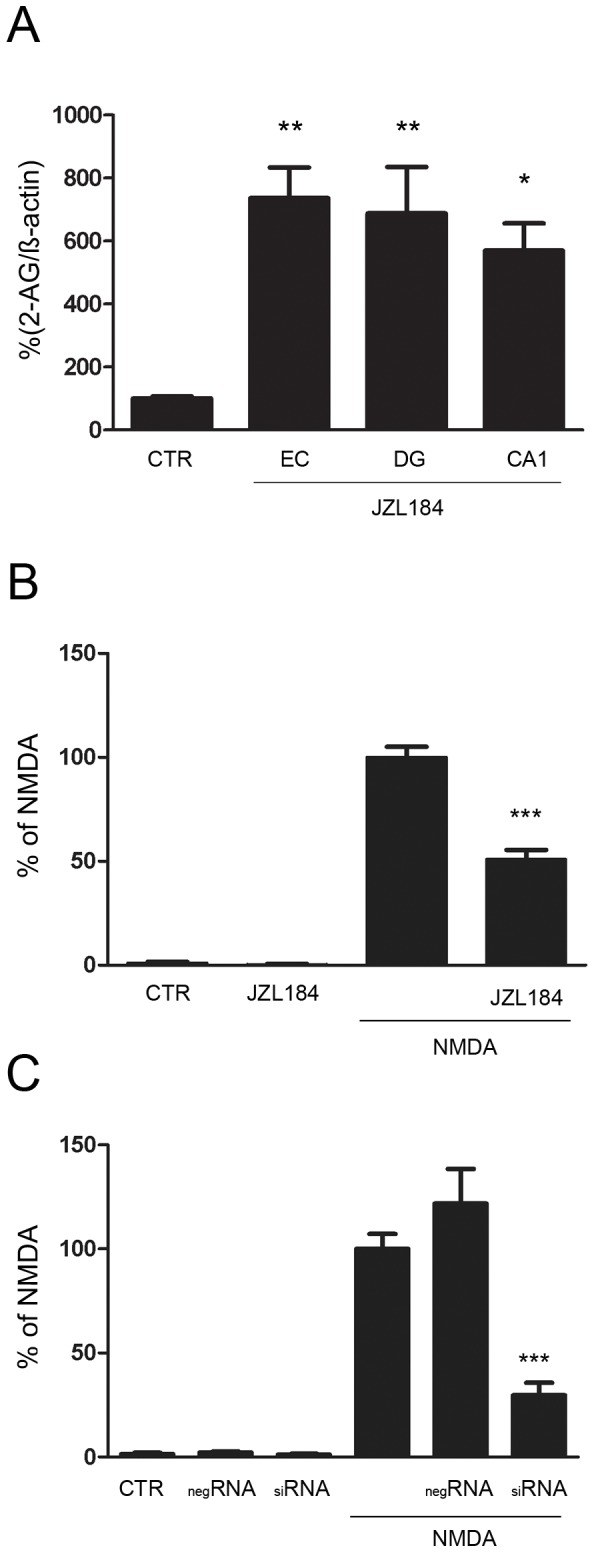
Inhibition of MAGL by JZL184 and siRNA exerted neuroprotective effects. (A) OHSC were treated for 4 h with JZL184 (1 µM) at 6 div. 2-AG levels were significantly elevated in all investigated (EC, DG, CA1) regions compared to the respective control regions of untreated OHSC. (B) Decreased neuronal cell death was seen in excitotoxically lesioned OHSC treated with JZL184 compared to lesioned OHSC treated with NMDA alone. (C) MAGL knock down by siRNA reduced the number of degenerating neurons after NMDA-lesion. (n>3, * = p<0.05, ** = p<0.01, *** = p<0.001).

After JZL184 (1 µM) application for 4 h 2-AG levels increased up to 35–45 ng/ml in OHSC and were significantly elevated in all investigated regions (EC, 736%; p<0.01; DG, 688%; p<0.01, CA1, 570%; p<0.05, Fig. 5A).

### Inhibition of MAGL by JZL184 and Magl knock-down using siRNA exerted neuroprotective effects after excitotoxic lesion

Non-lesioned control OHSC exhibited a good neuronal preservation with almost no PI positive neuronal nuclei. All conditions were normalized against NMDA and given in percent values (Fig. 5B). The application of 1 µM JZL184 did not led to any significant alterations within the number of PI positive neuronal nuclei as compared to CTR (JZL184, 0.24%, p>0.05). Treatment of OHSC with 50 µM NMDA for 4 hours at 6 div strongly increased the numbers of PI positive neuronal nuclei (NMDA, 100%, p<0.001 vs. negative CTL) as compared to CTR. Application of 1 µM JZL184 to NMDA-lesioned OHSC led to a significant reduction of PI positive neuronal nuclei as compared to positive OHSC treated with NMDA alone (NMDA+1 µM JZL184, 50.97%; p<0.05 vs. NMDA, Fig. 5B).

Non-lesioned OHSC treated with negRNA or siRNA (negRNA, 0.28% siRNA, 0.28%) exhibited no different neuronal preservation as compared to control OHSC with almost no PI positive neuronal nuclei (CTL, 0.26%) in the DG. Treatment of OHSC with 50 µM NMDA for 4 hours at 6 div strongly induced the numbers of PI positive neuronal nuclei (NMDA, 100%, p<0.001 vs. CTL) as compared to untreated CTL. Application of negRNA to NMDA-lesioned OHSC showed no changes (NMDA+negRNA, 121.83%; p>0.05 vs. NMDA), but the application of siRNA led to a significant reduction of PI positive neuronal nuclei compared to OHSC treated with NMDA alone (NMDA+siRNA, 33.45%; p<0.05 vs. NMDA, Fig. 5C).

## Discussion

Primary neurological insults are worsened by secondary accumulation of harmful mediators leading to delayed tissue damage and finally to persistent clinical and social disabilities for patients [32]. Therefore, one aim in current neuroscience research is the identification of biological mechanisms by which the protective properties of intrinsic brain mechanisms can be amplified with the final goal to develop clinical tools against secondary neuronal damage. 2-AG is commonly accepted as a beneficial mediator and an important member of the eCB system, well known to protect neurons after neuronal insults mainly by retrograde signaling finally leading to decreased neuronal excitation [4], [33], [34], [35]. Since the cell-type specific and temporal regulation of 2-AG during secondary neuronal damage in the brain was still fragmentary, we chose two different neuronal lesion models to investigate whether the different areas within the hippocampal formation would respond with a distinct time dependent and area specific 2-AG up-regulation after low and high concentrations of NMDA or PPT.

The EC showed no detectable changes in 2-AG levels after low or high NMDA concentrations. The DG however showed enhanced 2-AG levels 24 hours after the lesion with high NMDA concentrations. Interestingly, low NMDA doses were sufficient to enhance 2-AG significantly in the CA1 region. Being the region with the highest density of NMDA receptors the CA1 region is the most sensitive region to excitotoxic lesion [36]. Evidence exists that the density of the 2-AG hydrolyzing enzyme, MAGL, is low on the axon terminals of mossy cells and DAGL, the 2-AG synthesizing enzyme, was shown to be abundant at the Schaffer collateral CA1 region of the hippocampal formation [37], [38], [39]. This strengthens the relevance of 2-AG in the CA1 region during neuronal stress. Enhanced 2-AG response to low NMDA concentrations might be a possible mechanism to counteract the insult in the more susceptible CA1 region. Stimulation of the Schaffer collateral synapses shown by Stella et al. led also to an endogenous 2-AG release in rat OHSC [40] and also short-term synaptic depression was consistently enhanced by genetic inhibition of MAGL [41], [42] supporting the present data.

CNS pathologies like stroke involve lesion of long range projections and a wide range of temporal cellular reorganization. We focused on intrinsic 2-AG regulation after a model of long range projection damage, the perforant pathway transaction in OHSC. This model was described in detail recently [31] allowing clear differentiation of anterograde (DG), retrograde (EC) and more distant area (CA1) changes induced by axonal injury of long range projections without leading to neuronal cell death. At the direct axonal lesion site no changes of 2-AG were observed. However, at the site of anterograde (dendritic) lesion a maximum peak was seen 24 hours post lesion. We could clearly show that axonal degeneration resulted in a moderate 2-AG increase at the target area and had no further effects on 2-AG regulation in the CA1 region, postulating the involvement of 2-AG regulation in dendtitic re-organisation.

To assess the involvement of 2-AG in dendritic reorganization we investigated the regulation of the main synthesizing (DAGL) and hydrolyzing (MAGL) enzymes of 2-AG. *Dagl* mRNA remained unchanged after PPT in the EC and was increased 24 hpl in the DG, whereas *Magl* mRNA remained at control levels. DAGL^−/−^ mice show reduced 2-AG levels and it was found that DAGL is functionally involved in neurogenesis [43], [44]. Increased *Dagl* mRNA levels might therefore explain the enhanced 2-AG levels of the anterograde lesion site as suggested by a previous spinal cord study [45]. In our study the investigation of DAGL protein levels over time surprisingly revealed a decreased DAGL protein expression 24 hours after PPT.

A possible explanation for enhanced *Dagl* mRNA expression and increased 2-AG levels together with decreased DAGL protein expression might be ubiquitination of DAGL as reported in development and axonal growth [20], [49]. Ubiquitination is common among proteins involved in signaling events, where rapid regulation is important,. Very recently two enzymes (Phospholipase D1, Cyclooxygenase-1 (COX-1)) involved in arachidonic acid regulation were shown to be ubiquitinated [46], [47]. Intra-cellular calcium concentration was responsible in human embryonic kidney 293 cells for ubiquitinated COX-1 degradation [46]. The fast turnover of proteins via the ubiquitin–proteasome system is a fundamental mechanism for cells to control signal transduction, cell cycle regulation and transcription [48], [49]. Another hypothesis might be the reflectory increase in *Dagl* mRNA in response to decreased DAGL protein levels. To evaluate this hypothesis additional time points of investigation between 12 hpl and 24 hpl will be needed.

To clarify the regulation of DAGL and MAGL more precisely, we investigated their cellular distribution in neurons, microglia and astrocytes under control and LPS- stimulated conditions in OHSC as well as in primary cell cultures. DAGL was strongly found in neurons and was only weakly present in astrocytes of control OHSC. In primary neuronal cultures we found DAGL colocalized with Vglut1 at pre-synapses confirming previous reports in cerebellar, hippocampal and striatal neurons showing DAGL at the dendritic spines in the CA1 subfield, linked to the intracellular surface of the plasma membrane [38], [39], [50], [51]. In the present study, we found a re-organization of DAGL immunoreaction especially after PPT towards the area of dendritic reorganization. Interestingly, the DAGL immunoreaction increased in astrocytes after both, PPT and excitotoxicity. Reactive GFAP positive astrocytes have been found to produce 2-AG and express DAGL in a valuable amount [52] either in vitro or in vivo in the epicenter of spinal cord injury [45]. In addition, our findings are in line with a previous descriptions of intense 2-AG production after stimulating isolated astrocytes with high ATP concentrations [53].

Microglia however did not show DAGL immunoreaction in OHSC, but in primary microglia culture. Interestingly, DAGL immunoreacitvity was not observed in microglia after spinal cord injury [45], although microglia have been shown to produce important amounts of 2-AG in vitro [54], [55].

The distribution of the degrading enzyme MAGL displayed a surprising pattern. Most studies focused on neuronal expression of MAGL, however the cellular expression of MAGL during NMDA lesion and PPT showed a low MAGL expression in astrocytes compared to controls by immunohistochemistry. It has previously been reported that astrocytes express the degrading enzyme MAGL [56], [57]. Here, a clear MAGL immunoreaction in microglia was not found in OHSC but in microglial primary cell cultures. Recent studies suggest that the ABHD12 enzyme, highly expressed in microglia accounts for approx. 9% of total brain 2-AG hydrolysis [58]. ABHD12 was suggested as adequate MAGL analogue for microglia as well as in related cell types of peripheral tissues [59]. The different distribution of DAGL and MAGL immunoreactivities in different cellular states suggests a highly defined expression pattern depending on the activation state of the cells. Therefore, our findings imply a qualitatively different contribution of 2-AG signaling to distinct lesion models as described for specific synapses and microcircuits.

CB_1_ and DAGL are evolutionary closely associated, stating 2-AG as the endogenous ligand for CB_1_ [10], [60]. Interestingly, we observed no alteration in the amount of CB_1_ protein in the DG 24 hpl, the primary target of 2-AG [31]. Recent reports found a decrease in CB_1_ by internalization or downregulation after prolonged 2-AG stimulation in coronal brain section by [^35^S] GTPγS binding [61]. The absence of MAGL led to compensatory desensitization of the CB1 receptor in the brain as CB_1_ receptor density and functional responses were attenuated [61]. Here, we used JZL184 to induce an up-regulation of intrinsic 2-AG levels and observed increased 2-AG levels in all investigated regions. It is reported that chronic inactivation of MAGL by JZL184 dramatically reduced 2-AG break-down, that led to a 10-fold increase of 2-AG levels in the brain as also shown in the present study [58], [62]. However, JZL184 was less potent in the CA1 region than in the DG or the EC, supporting the lower occurrence of MAGL in the CA1 region.

To further demonstrate that intrinsic up-regulation of 2-AG, clearly accounts for neuronal protection, we treated NMDA lesioned OHSC with either JZL184 or MAGL siRNA and observed decreased neuronal damage in the granular cell layer of the dentate gyrus under both conditions. Other studies reported neuroprotective properties of 2-AG in neuronal excitotoxic models and other cellular settings by using exogenous 2-AG [11], [63]. However, in vivo models of closed head injury and focal ischemia in mice elevated 2-AG levels were found after the lesion [4], [64]. The early onset of 2-AG level up-regulation might be provoked by the blood-brain barrier (BBB) disruption as 2-AG levels were shown to reduce BBB permeability 1–4 hours after brain injury [64], [65].

In summary, 2-AG is intrinsically involved in general secondary damage regulation and strongly responds to excitotoxic and axonal damage in OHSC as a neuroprotective mediator. Furthermore the DAGL and MAGL dependent regulation of 2-AG is a dynamic interplay mainly of astrocytes and neurons embedded in time and cellular organization and involved in dendritic reorganisation.

## Supporting Information

Table S1 **Sequences of the used primers.**
(TIF)

Figure S1 **Specificity test for antibodies against MAGL by Western blot analyses.** (A) The antibody against MAGL showed two immunoreactive bands of about 35 kDa (1). Both bands were blocked by use of the respective blocking peptide (2). (B) MAGL fluorescent staining and MAGL fluorescent staining after preincubation with the respective peptide for 1 h. Bar = 50 µm.
(TIF)
