# Supplementary figures and images for: Intrinsic Up-Regulation of 2-AG Favors an Area Specific Neuronal Survival in Different In Vitro Models of Neuronal Damage
Source: PLoS One. 2012 Dec 20;7(12):e51208. doi: 10.1371/journal.pone.0051208 (PMC3527460; doi:10.1371/journal.pone.0051208)

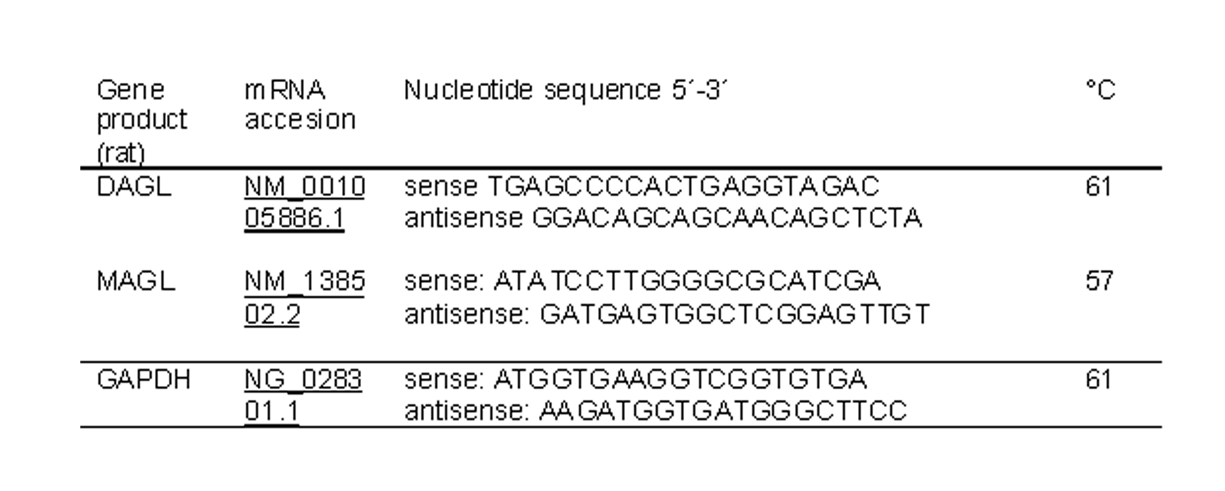

Supplement: Table S1 — Sequences of the used primers. (TIF) [file pone.0051208.s001.tif]

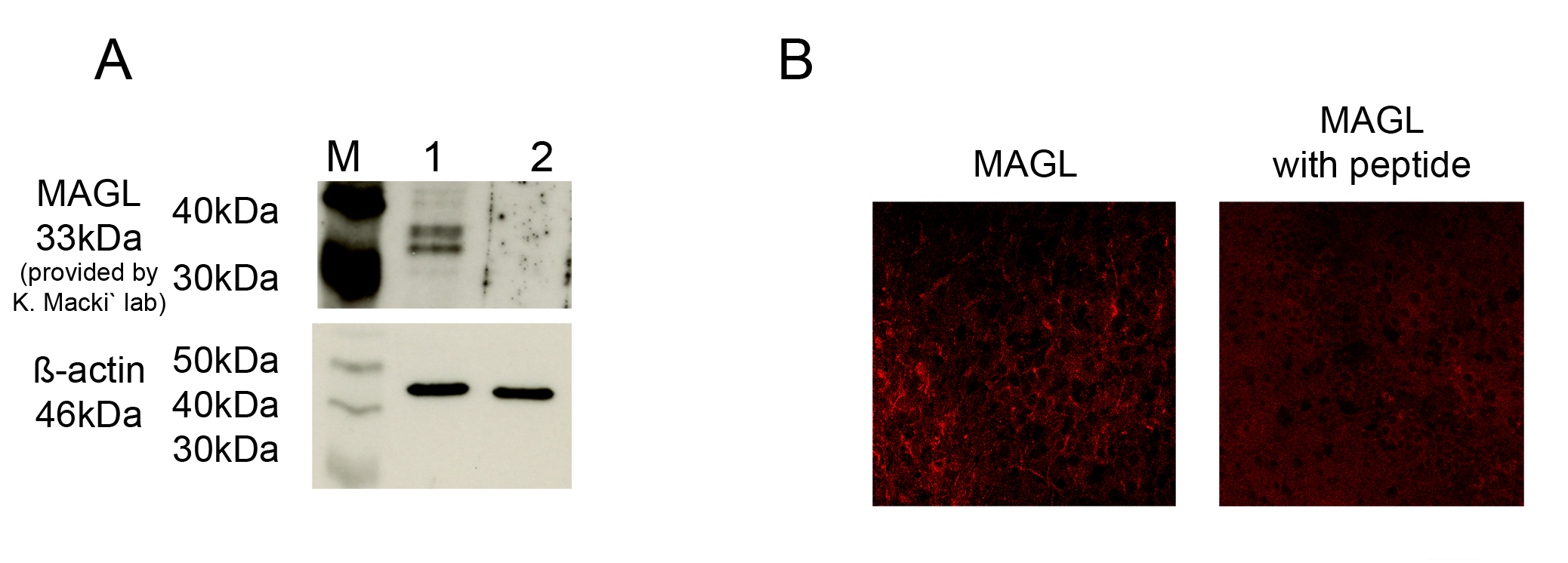

Supplement: Figure S1 — Specificity test for antibodies against MAGL by Western blot analyses. (A) The antibody against MAGL showed two immunoreactive bands of about 35 kDa (1). Both bands were blocked by use of the respective blocking peptide (2). (B) MAGL fluorescent staining and MAGL fluorescent staining after preincubation with the respective peptide for 1 h. Bar = 50 µm. (TIF) [file pone.0051208.s002.tif]
